# Back in 3D—a report on Genome Informatics 2022

**DOI:** 10.1186/s13059-023-02870-8

**Published:** 2023-03-28

**Authors:** Iman Hajirasouliha, Stefan Semrau

**Affiliations:** 1 Institute for Computational Biomedicine, Englander Institute for Precision Medicine, The Meyer Cancer Center, Department of Physiology and Biophysics, Weill Cornell Medicine of Cornell University, New York City, NY 10021 USA; 2 Leiden Institute of Physics, Leiden University, Leiden, The Netherlands; 3 Computational Biology, New York Stem Cell Foundation, New York City, USA

## Abstract

The annual Genome Informatics conference was held at the Wellcome Genome Campus on September 21–23, 2022. The conference covered a remarkable range of topics of which we highlight a few in this report.

## Introduction

For the first time since the beginning of the COVID pandemic in 2019, it was finally possible to hold the Genome Informatics conference in person. The joy of getting together and discussing science face-to-face again was palpable among the participants, and the luscious green Wellcome Genome Campus close to a burbling river Cam was the perfect backdrop. Catching up with old friends and making new connections around the globe continued into the late hours, partially thanks to the proprietor of the Red Lion Inn, who kept his establishment open for some thirsty bioinformaticians. The many excellent presentations and poster contributions provided ample points of discussion and new ideas needed to be worked out.

### Obesity—it is not about willpower

After jokingly confessing to being intimidated by three-dimensional space after so much time behind a two-dimensional screen during the pandemic, Cecilia Lindgren gave an impressive opening keynote presentation. Her research of the “underdog” phenotype obesity spans the gamut from finding causal genetic variants to verifying potential functions in cellular models and in vitro screens. To the possible demur of some graduate students, Lindgren cautioned that quality control is crucial for any GWAS study and advised to spend at least 70% of your time on it. Among many other new associations, her team identified neuroendocrine pathways that likely modulate sensations of satiety and hunger, as well as a transcription factor that regulates the development of adipocytes from mesenchymal stem cells. Following up on the latter, the lab set up collaborations to reveal the link between obesity-related cellular phenotypes in adipocytes and genetic variants. Lindgren’s own work and the International Common Disease Alliance she co-organizes are prime examples of the large-scale, collaborative team science that has become so important in our field.

### Islands of significance

Reflecting the diversifying arsenal of experimental methods, a whole session of Genome Informatics was dedicated to single-cell and spatial omics for the first time this year. Interest in spatial omics has been growing quickly while methods are still being refined and new data analysis tools are being developed.

Helder Nakaya presented a new tool for locating the expression of gene sets in space, spatial enrichment gene set analysis or SEGA. (We keep our fingers crossed that the corporation of the same name does not scan the bioinformatics literature for cases of trademark infringement.) To avoid false positives from areas with globally high expression, SEGA calculates the significance of gene set enrichment for each measured location. The result of this analysis can be visualized as a partially flooded landscape, where the water level corresponds to significance. As examples for “islands of significance,” Nakaya showed gene sets of tumor-infiltrating CD8 T cell subtypes. Downstream analysis of the distances between the islands might reveal new insights into T-cell biology.

In the same session, Sarah Teichmann, whose lab is one of the driving forces behind the Human Cell Atlas, reviewed some of her efforts to integrate single-cell and spatial omics data of the human heart. Highlighting once more the collaborative nature of our research field, Teichmann’s lab teamed up with a cardiac anatomist to complement and refine gross tissue anatomy with spatial gene expression data on the micrometer scale. Teichmann’s team focused specifically on the pacemaker cells, which stimulate the heartbeat and had not been characterized at the single-cell level in humans. This work predicted a number of drugs not meant to target the cardiovascular system nevertheless to affect pacemaker cells.

A common and unsolved problem in the single-cell omics community was addressed by Sindri Antonsson. Often, multiple single-cell data sets must be integrated without confusing uninformative (technical) batch effects and interesting biological variability. Antonsson analyzed several common data integration methods and found that some of them introduce serious artifacts. When applied to two data sets created by random sampling from the same population of cells, some methods significantly distorted the distance of cells in gene expression space and even shuffled cells between distinct cell types. Antonsson’s presentation was an important reminder that we must always consider the limits of data analysis techniques and that careful benchmarking studies are extremely valuable.

### Nothing as practical as a good theory

Unique to this year’s meeting, some of the speakers presented very algorithmic and theory-heavy work. In particular, Nicola Prezza presented novel theoretical results on the pattern-matching problem on index data structures for multiple sequences. His results were accepted for presentation at SODA 2022, a top theoretical computer science venue. Dmitrii Meleshko, a Ph.D. student from Hajirasouliha’s lab, one of the authors of this report, presented a new version of his Blackbird algorithm. This algorithm assembles structural variants using hybrid synthetic long reads (SLR) and low-coverage long reads. By modifying the SPAdes assembly graph, Blackbird accommodates both technology platforms.

Ryan Wick gave a very entertaining and engaging presentation during which he also unexpectedly made his tool publicly available on GitHub. The tool he presented was developed to build phylogenies of bacterial genomes assuming they are recombination free. Wick deservedly won the prize for the best short presentation of the meeting. Camille Marchet from the CNRS in France presented a tool named PAC, which is based on a novel approximate membership query data structure. The tool she presented is useful for querying collections of sequence datasets. PAC uses Partitioned Aggregated Bloom Comb-Trees and offers significant improvement in construction time when compared with existing tools.

### Challenges and opportunities

The Genome Informatics meeting was concluded with a keynote talk by Peer Bork. Among other topics, he discussed the challenges of pipeline development for metagenomics. The use of many different tools for metagenome assembly has led to redundant, overlapping, and in some cases chimeric genomes. He also highlighted strain-resolved assembly of metagenomes as another unsolved problem. Bork’s keynote was the final highlight in a string of excellent talks both from invited speakers and selected from contributed abstracts. In several talks, the presenters discussed the current challenges of handling large genomics and metagenomics datasets and databases. With the explosive growth of genomic datasets showing no signs of letting up, we anticipate hearing more about algorithmic and machine-learning developments focusing on big data at next year’s meeting. In conclusion, we think that Genome Informatics 2022 was a success, with many face-to-face interactions that have strengthened our community and hopefully led to new ideas and collaborations.

